# Hyperbaric Oxygen Therapy (HBOT) in the Treatment of Autism Spectrum Disorders

**DOI:** 10.1192/j.eurpsy.2026.11803

**Published:** 2026-07-31

**Authors:** W. Wójtowicz, K. Jankowska, N. Świderski, M. Iwan, A. Bielecka-Wajdman

**Affiliations:** 1 Student Scientific Society at the Department of Pharmacology; 2 Department of Pharmacology, Medical University of Silesia, Katowice, Poland

## Abstract

**Introduction:**

Autism spectrum disorder (ASD) represents a heterogeneous group of neurodevelopmental conditions with an etiology involving complex interactions between environmental, genetic, and neurobiological factors. Advances in understanding the pathogenesis of ASD, together with the rising prevalence of diagnoses, highlight the need for more effective therapeutic approaches. One potential adjunctive intervention is hyperbaric oxygen therapy (HBOT). Its proposed mechanisms of action in ASD include anti-inflammatory effects, modulation of neuroplasticity, and a possible influence on oxidative stress. Current studies suggest that HBOT may improve certain behavioral and cognitive domains in individuals with ASD, such as speech, communication, and psychosocial functioning. However, findings remain inconsistent, partly due to potential adverse effects of HBOT, including barotrauma and oxygen toxicity.

**Objectives:**

The aim of this study is to evaluate current evidence on the efficacy and safety of HBOT as a potential adjunctive treatment for ASD, with particular emphasis on its effects on neuroinflammatory processes, neuroplasticity, oxidative stress, and the behavioral and cognitive functioning of patients.

**Methods:**

This work presents a review of 45 experimental studies published between 2012 and 2025. The analysis was conducted using PubMed and Google Scholar databases, with search terms including: autism, hyperbaric oxygen therapy (HBOT), autism spectrum disorder (ASD), neuroinflammation, and complementary therapies.

**Results:**

The literature review indicates that the beneficial effects of HBOT are primarily associated with its anti-inflammatory properties, including the downregulation of pro-inflammatory cytokine gene expression and the promotion of neural tissue regeneration. Its impact on reducing oxidative stress and mitochondrial dysfunction has not yet been conclusively demonstrated and requires further investigation. Available functional studies also do not provide sufficient evidence to recommend HBOT as a standard therapy for children with ASD. While improvements have been observed in certain domains (e.g., psychosocial functioning, imitation, imagination), deterioration in others (e.g., response to commands) has also been reported.

**Conclusions:**

HBOT warrants further investigation in the context of ASD, as it demonstrates potential anti-inflammatory effects, modulation of neuroplasticity, and influence on oxidative stress and mitochondrial function—mechanisms implicated in ASD pathogenesis. Moreover, available findings suggest possible improvements in selected cognitive and behavioral functions. However, inconsistencies in the current evidence and methodological limitations of existing studies underscore the need for further well-designed clinical trials.

**Disclosure of Interest:**

None Declared

